# Development of a silicon limitation inducible expression system for recombinant protein production in the centric diatoms *Thalassiosira pseudonana* and *Cyclotella cryptica*

**DOI:** 10.1186/s12934-017-0760-3

**Published:** 2017-08-17

**Authors:** Roshan P. Shrestha, Mark Hildebrand

**Affiliations:** Scripps Institution of Oceanography, University of California San Diego, La Jolla, CA USA

**Keywords:** Diatom, *Thalassiosira pseudonana*, *Cyclotella cryptica*, Transformation, Inducible promoter, Silicon transporter, Silicon-limitation, Protein expression, Fatty acid synthesis inhibition

## Abstract

**Background:**

An inducible promoter for recombinant protein expression provides substantial benefits because under induction conditions cellular energy and metabolic capability can be directed into protein synthesis. The most widely used inducible promoter for diatoms is for nitrate reductase, however, nitrogen metabolism is tied into diverse aspects of cellular function, and the induction response is not necessarily robust. Silicon limitation offers a means to eliminate energy and metabolic flux into cell division processes, with little other detrimental effect on cellular function, and a protein expression system that works under those conditions could be advantageous.

**Results:**

In this study, we evaluate a number of promoters for recombinant protein expression induced by silicon limitation and repressed by the presence of silicon in the diatoms *Thalassiosira pseudonana* and *Cyclotella cryptica*. In addition to silicon limitation, we describe additional strategies to elevate recombinant protein expression level, including inclusion of the 5′ fragment of the coding region of the native gene and reducing carbon flow into ancillary processes of pigment synthesis and formation of photosynthetic storage products. We achieved yields of eGFP to 1.8% of total soluble protein in *C. cryptica*, which is about 3.6-fold higher than that obtained with chloroplast expression and ninefold higher than nuclear expression in another well-established algal system.

**Conclusions:**

Our studies demonstrate that the combination of inducible promoter and other strategies can result in robust expression of recombinant protein in a nuclear-based expression system in diatoms under silicon limited conditions, separating the protein expression regime from growth processes and improving overall recombinant protein yields.

**Electronic supplementary material:**

The online version of this article (doi:10.1186/s12934-017-0760-3) contains supplementary material, which is available to authorized users.

## Background

A number of algal systems have been developed to produce nuclear-encoded recombinant proteins. In some algae, nuclear expression can be problematic due to epigenetic transgene silencing, in which an introduced gene loses its ability to be expressed [1]. In this case, several options are possible. The first relies on fusing the desired protein to a selectable marker, and maintaining selection conditions during expression [2, 3]. Incorporating a viral 2A amino acid sequence between the two fusion partners results in two separate protein products, so the desired recombinant protein can be generated. In one case in *Chlamydomonas reinhardtii*, this approach was used with a mutagenized cell line to reduce methylation that contributed to gene silencing [4]. Secretion of recombinant proteins into the growth media could be an alternative solution to problems in protein expression, including aggregation, incorrect folding or toxicity [5]. A chloroplast expression system is another method [6]. Although there are many advantages to chloroplast expression [7–9], it is not suitable when recombinant proteins need post translational modifications, and in obligate phototrophic organisms that cannot grow on an external source of carbon, accumulation of a large quantity of a foreign protein could be detrimental to photosynthesis. The converse has been demonstrated; expression of recombinant GFP in the *C. reinhardtii* chloroplast was negatively affected by high light or low culture density [10], perhaps because of a drain on protein synthesis capability to replace photosynthetic protein turnover. Heterotrophic cultivation can alleviate this problem, but addition of extracellular carbon increases production cost and the risk of contamination. Nuclear-based expression can offer other advantages, for example, targeting of expressed protein to different intracellular compartments. In plants, protein expression yields are generally substantially improved when targeting is to the endoplasmic reticulum (ER), because of increased protein folding capability and stability in that compartment [11, 12]. There are algal species such as diatoms that do not suffer from epigenetic gene silencing [13], and developed algal nuclear expression systems include species of diatoms, green, and red algae, where a number of different proteins, including antigens and antibodies, and those capable of synthesizing bioplastics, were successfully expressed, as reviewed in [6, 14].

Transcriptional control elements such as the promoter exert a major control over gene transcript levels and ultimately protein production. Several constitutive promoters have been used to drive protein expression in algae [15]. Despite their advantages for production of proteins such as selectable markers or reporter proteins, they are not always desirable when expressing high levels of a protein for commercial purposes. For example, overproduced proteins can suppress host cell growth and metabolism by dominating the translation process and draining cellular biomolecules and energy from essential metabolic and growth processes. In fact, in the most highly developed recombinant protein expression systems, such as bacteria and yeasts, conditions in which cellular growth is blocked or severely retarded are desirable because they facilitate extra metabolic capability and energy flow into recombinant protein expression [16, 17]. Moreover, toxic proteins can kill the host organism or negatively affect growth [18]. In both scenarios, the use of inducible promoters in expression vectors is beneficial. These could be turned on or off via simple manipulations such as changing a nutrient concentration in the media or adding a chemical compound, and thus can control the timing of protein expression, for example, during specific phase of the life or cell cycle, or under growth arrest conditions. Thus far, only a few inducible promoters are available for algal expression. The nitrate reductase (NR) promoter, which enables expression in the presence of nitrate or absence of nitrogen and repression by ammonium, is the best available inducible element. The NR promoter was developed for the pennate diatom *Cylindrotheca fusiformis* [19], and subsequently adapted for other diatoms including *T. pseudonana* [20] and *Phaeodactylum tricornutum* [21]. This promoter has been used to control expression of proteins making bioplastics [22] and IgG antibodies [23] in *P. tricornutum*. The NR promoter has been used in other algae, including *Chlorella ellipsoidea, C. vulgaris, Dunaliella salina* and *C. reinhardtii* [24–27]. In addition, a few other inducible promoter systems, which can be induced by chemicals, physical factors such as heat, and deficiency of certain elements and nutrients have been reported. The *Cpx1 and Cyc6* genes of *C. reinhardtii* are one of a few chemically regulated genes, the expression of which were elevated under copper deficiency or addition of nickel or cobalt in the medium [28]. The low CO_2_-inducible *CAH1* promoter and heat inducible promoters of heat shock proteins such as *hsp70A* of *C. reinhardtii* were shown to be useful without adding any toxic heavy metals [29, 30]. The arylsulphatase promoter of *Volvox carteri* was shown to be a useful inducible promoter under sulfur starvation conditions [31, 32].

All of the published inducible promoters to date are inducible under conditions of cell growth, or involve conditions that are detrimental to the cell. Because the most highly developed recombinant protein expression systems in other organisms utilize conditions in which metabolic capacity and energy flow into recombinant protein expression is maximized [16, 17], we wanted to develop a similar capability in diatoms. Under silicon limited conditions, cell cycle progression and growth in diatoms is blocked, but other aspects of cellular metabolism are not negatively affected [33, 34]. We anticipate that these conditions will be amenable to recombinant protein expression. To this end, we report on the development of inducible expression systems for diatoms based on promoters driving expression of silicon transporters (SITs) and other genes concomitantly expressed with SITs in silicon (Si)-rich and Si-deficient media. The SITs are downregulated under sufficient silicic acid concentrations—a condition where silicic acid uptake occurs primarily by diffusion, and are highly upregulated during silicon starvation [35, 36]. This system allows separation of the cell growth phase and the recombinant protein production phase [37], which enables channeling of energy and metabolic capacity normally used for cell cycle progression.

## Results

### Identification of genes whose transcripts are upregulated by Si limitation

**Fig. 1:**
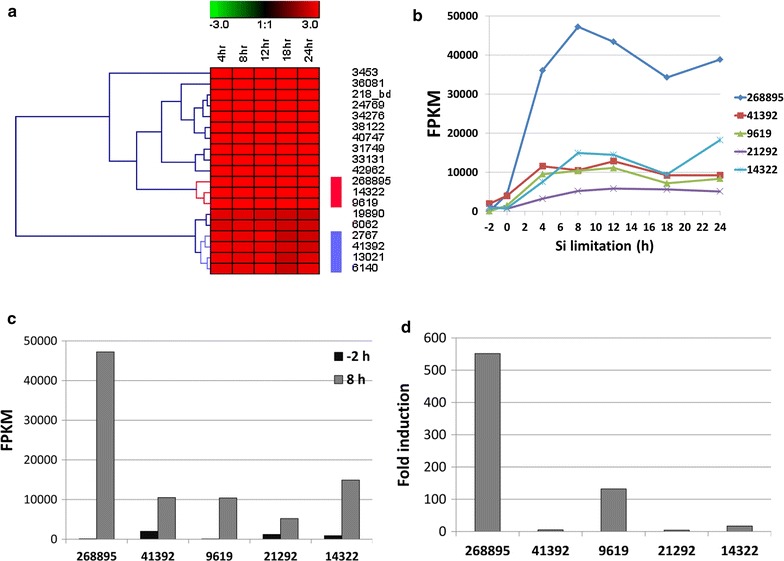
Identification of *T. pseudonana* genes induced by silicon limitation and repressed by silicon presence. **a** Hierarchically clustered mRNA expression profile from Affymetrix microarrays after silicon starvation showing SIT1 and SIT2 clusters [39].* Columns* correspond to log2ratio (fold change) of the time points (4, 8, 12, 18 and 24 h) relative to 0 h of silicon starvation. The intensities of the *colors* indicate the magnitude of up regulation (*red*) and down regulation (*green*). *Black* indicates no change. The TpSIT1 and TpSIT2 clusters are indicated by the *red* and *blue bars*, respectively. **b** Transcript abundance levels of genes in TpSIT1 and 2 clusters determined by RNAseq [39]. FPKM values are plotted, the time course of Si limitation (0–24 h) includes a time point at −2 h, which is Si replete conditions. **c** FPKM values of genes in Si replete medium at −2 and 8 h after Si starvation. **d** Fold induction of genes at 8 h relative to −2 h

*Thalassiosira pseudonana* silicon transporter TpSIT1 (Thaps3_268895) and TpSIT2 (Thaps3_41392) mRNA levels are upregulated by Si limitation and downregulated under Si replete conditions, with TpSIT1 having a higher induction than TpSIT2 [35, 38]. Using whole genome microarray data from *T. pseudonana* [39], we identified other genes whose mRNA expression patterns clustered with TpSIT1 and 2 (Fig. 1a), and designated the expression elements promoting them silicon starvation inducible promoters (SSIPs). In a separate experiment, RNAseq transcriptome sequencing [39] was done on a time course of Si limitation, which included a time point 2 h prior to placing cells in Si-free medium, corresponding to exponential growth in Si replete conditions. All clustered genes were induced during Si limitation, but TpSIT1 was induced far higher than the others (Fig. 1b). In fact, mRNA for TpSIT1 was the second most abundant transcript in the entire genome under Si limiting conditions. Plotting FPKM (Fragments Per Kilobase of transcript per Million mapped reads) for the −2 h time point (Fig. 1c) showed that TpSIT1 had the second lowest RNA abundance in the cluster under uninduced conditions, but Fig. 1d shows that transcript levels were induced over 500-fold by 8 h Si limitation.

### SSIP promoters strongly induce protein expression during silicon deprivation

**Fig. 2:**
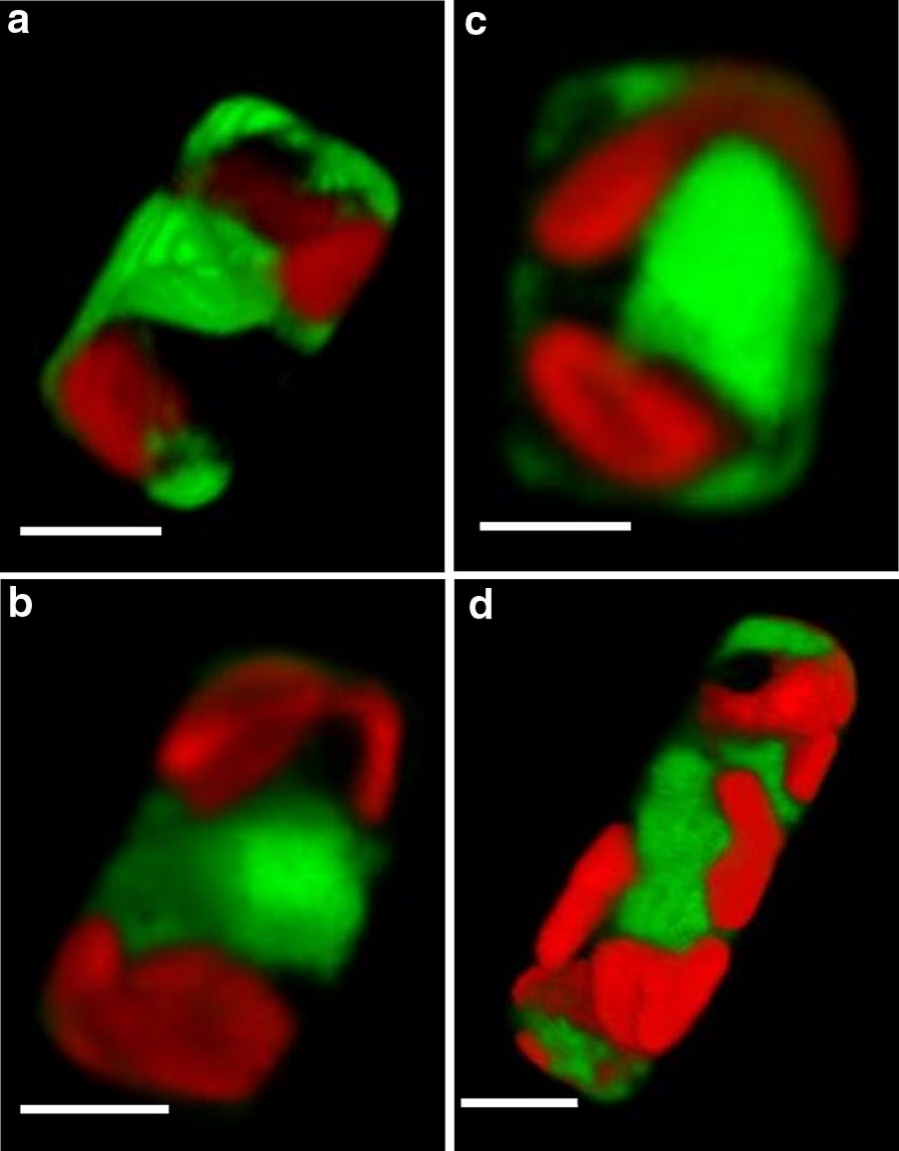
Expression of eGFP under control of the Si-inducible promoters of *T. pseudonana* and *C. cryptica* after 24 h of Si starvation showing cytoplasmic localization. **a** TpSIT1 (Thaps3_268895); **b** TpSIT2 (Thaps3_41392); 9619, **c** (Thaps3_9619); and **d** CcSIT1, (g10780.t1). *Scale bar* 2 µM

To initially evaluate the characteristics of the SSIPs with regard to protein expression, we constructed vectors expressing eGFP under control of their promoters for cytoplasmically targeted expression. Antibiotic-resistant transgenic clones were selected from agar plates, and we evaluated only the best GFP expressing lines by selecting clones with the brightest GFP fluorescence for further study. We also included a construct of a SIT gene from *C. cryptica*, CcSIT1_g10780.t1 to study expression in that species. Fluorescence microscopy revealed a high level of expression from all constructs under Si starvation, and eGFP occupied a large portion of the cytoplasm (Fig. 2).

Using imaging flow cytometry to evaluate large numbers of cells, we observed that not all cells in a clonal population expressed eGFP under Si-limiting conditions, and also detected a few cells expressing eGFP in Si-replete cultures. The lack of expression in all cells has been seen with other promoters under other culture conditions [40], so the phenomenon is common. These phenotypes are likely due to epigenetic effects. Because overall expressed protein yield depends on the level of expression per cell and the percentage of cells in the population that are expressing, we evaluated three parameters related to expression, the mean eGFP fluorescence per cell, the percentage of cells expressing detectable levels of eGFP, and the net expression of eGFP, which was the product of the mean expression times the percentage of expressing cells. It should be noted that because on the order of 20,000 cells are being evaluated in these and subsequent measurements, the standard error is miniscule, and error bars are not typically visible.

**Fig. 3:**
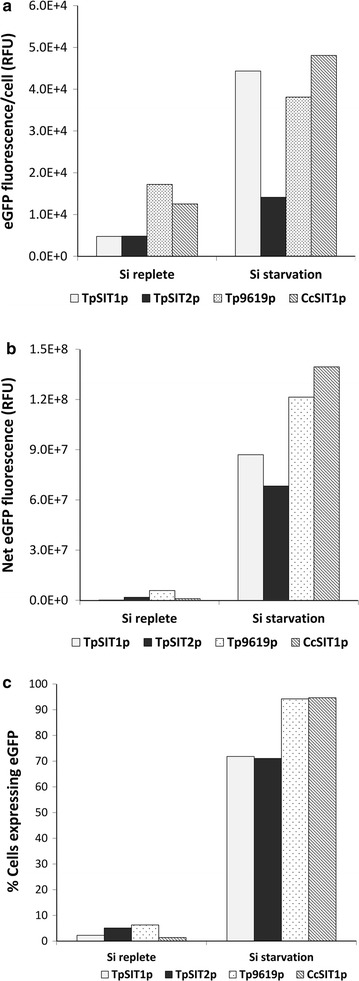
Induction level of *T. pseudonana* and *C. cryptica* SSIPs in Si-replete media and after 24 h of Si starvation as determined by imaging flow cytometry. **a** Mean eGFP fluorescence per cell. **b** Net eGFP fluorescence calculated as product of eGFP intensity/cell and no. of cells expressing eGFP. **c** Percentage of cells expressing eGFP. eGFP was measured with a 488-nm laser with 50 mw power output without a blocking filter. *Error bars* represent standard errors of mean (SEM), n = 20,000. TpSIT1p, Thaps3_268895; TpSIT2p, Thaps3_41392; Tp9619p, Thaps3_9619; and CcSIT1p, g10780.t1

Comparison of the SSIPs revealed different responses (Fig. 3). Variation was observed with the different promoters, but there was a consistent substantial increase in eGFP fluorescence and in the percentage of cells expressing under Si starvation (Fig. 3). Overall, at least 70% of the cells expressed eGFP after 24 h of Si starvation, and less than 6% of the cells expressed eGFP in Si-replete media. Amongst the *T. pseudonana* genes, SIT1 exhibited the highest gain in net expression, whereas Thaps3_9619 showed the highest absolute net expression. CcSIT1 also demonstrated a high degree of inducibility (Fig. 3).

### Constitutive promoters expressing under both Si-replete and Si-deplete condition

**Fig. 4:**
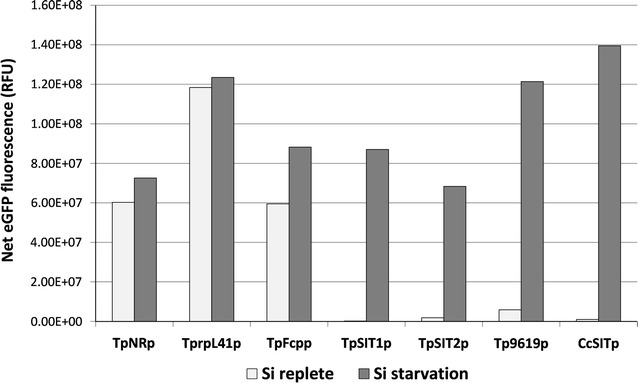
Comparison of eGFP expression driven by Si inducible and non Si inducible promoters in Si-replete media and after 24 h of Si starvation. The net eGFP fluorescence was calculated as product of eGFP intensity/cell and no. of cells expressing eGFP derived from imaging flow cytometry. To compensate for different levels of expression, eGFP was measured with a 488-nm laser with 100 mW power output without filter (TpNRp, TprpL41p, TpFcpp) and 30 mW power output after blocking saturation with neutral density filter 1.0 (TpSIT1p, TPSIT2p, Thaps3_9619p, and CcSITp). *Error bars* represent standard errors of mean (SEM), n = 20,000. *NR* nitrate reductase promoter, *rpL41p* ribosomal protein L41 promoter, *Fcpp* fucoxanthin chlorophyll a/c binding protein promoter

To evaluate the effect of silicon limitation on protein expression in general, we also analyzed expression of eGFP under the control of other promoters commonly used in diatoms, namely the nitrate inducible NR promoter, and constitutive promoters of fucoxanthin chlorophyll a/c-binding protein (fcp) and ribosomal protein rpL41. In all cases, net eGFP expression was increased under Si limitation conditions, with improvements of 1.04-fold for rpL41, 1.2-fold for NR, and 1.5-fold for fcp (Fig. 4). Interestingly, transcript levels only for fcp were induced under these conditions, both NR and rpL41were slightly reduced or there was no change (Additional file 1: Figure S1). This suggests that the improvement in yield is due to factors other than intrinsic capability to synthesize protein based on transcript abundance.

### Strategies to increase expression level of recombinant proteins: inclusion of the N-terminal region of corresponding genes

**Fig. 5:**
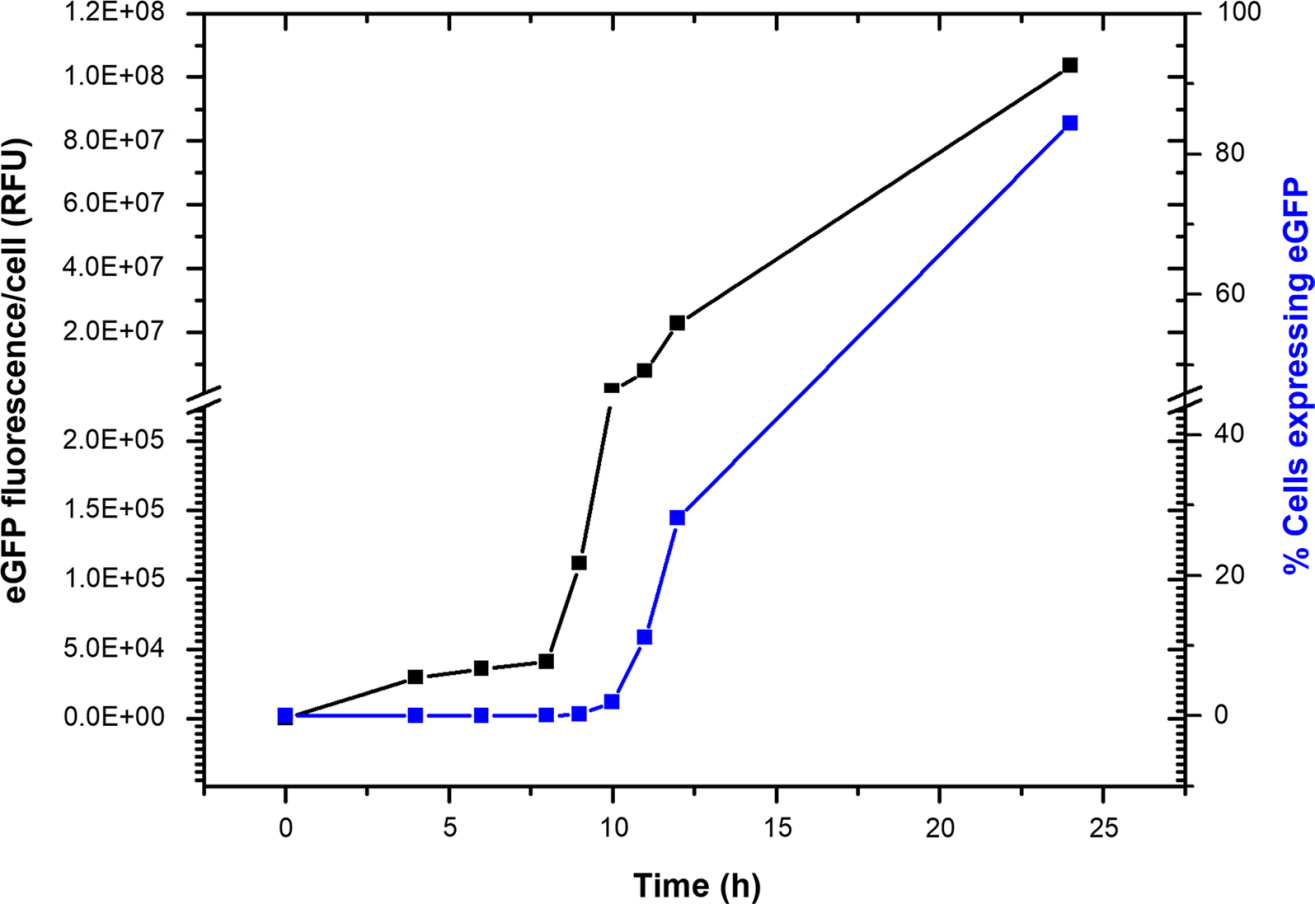
A time-course expression analysis of eGFP expression under control of TpSIT1 promoter in *Thalassiosira pseudonana*. Exponentially growing cells were transferred to silicon-deprived media (0 h) and expression of eGFP was followed by measuring eGFP fluorescence using an imaging flow cytometer (a 488-nm laser with 100 mW power output without a blocking filter). *Error bars* represent standard errors of mean (SEM), n = 20,000

Because TpSIT1 had the highest transcript level (Fig. 1) and most well-regulated eGFP expression response (Fig. 3) of all promoters, we focused on this promoter for subsequent evaluation and improvement of the protein expression system in *T. pseudonana*. An analysis of eGFP appearance under the control of the SIT1 promoter was performed using imaging flow cytometry to determine the timing of response of protein accumulation upon transfer of exponentially grown cells into Si-free medium. While most of the cell population was fluorescent by 24 h, some cells expressed eGFP as early as 6 h (Fig. 5), showing cellular heterogeneity. Hence, we used the 24 h time point to compare overall eGFP expression level in this study.

**Fig. 6:**
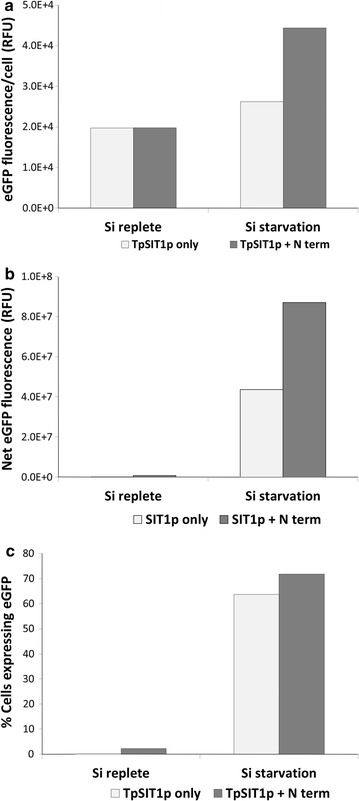
Effect of N-terminus presence (TpSIT1P + N term) or absence (TpSIT1p only) coding sequence of SIT1 on expression of eGFP. eGFP was expressed under the transcriptional control of TpSIT1 promoter. A 96-nt long 5’ coding sequence was cloned between promoter and eGFP (TpSIT1p + N term). eGFP was measured with a 488-nm laser with 50 mw power output without a blocking filter. Net eGFP fluorescence (**b**) = eGFP fluorescence/cell (**a**) x No. of cells expressing eGFP, depicted as % in (**c**). Error bars represent standard errors of mean (SEM), n = 20,000

We determined that eGFP had an unfavorable codon bias for *T. pseudonana* (Additional file 2: Figure S2), which could inhibit the translatability of the transcript. In attempt to enhance protein production with efficient translation initiation, we fused the 32-residues long N-terminal sequence of TpSIT1 gene (which has optimal codon usage—Additional file 2: Figure S2), to eGFP eliminating the amino-terminal codon bias of eGFP. We observed about twofold higher net expression of eGFP when theTpSIT1 N-terminus was present in comparison to eGFP alone (Fig. 6).

### Quantification of eGFP yield

**Fig. 7:**
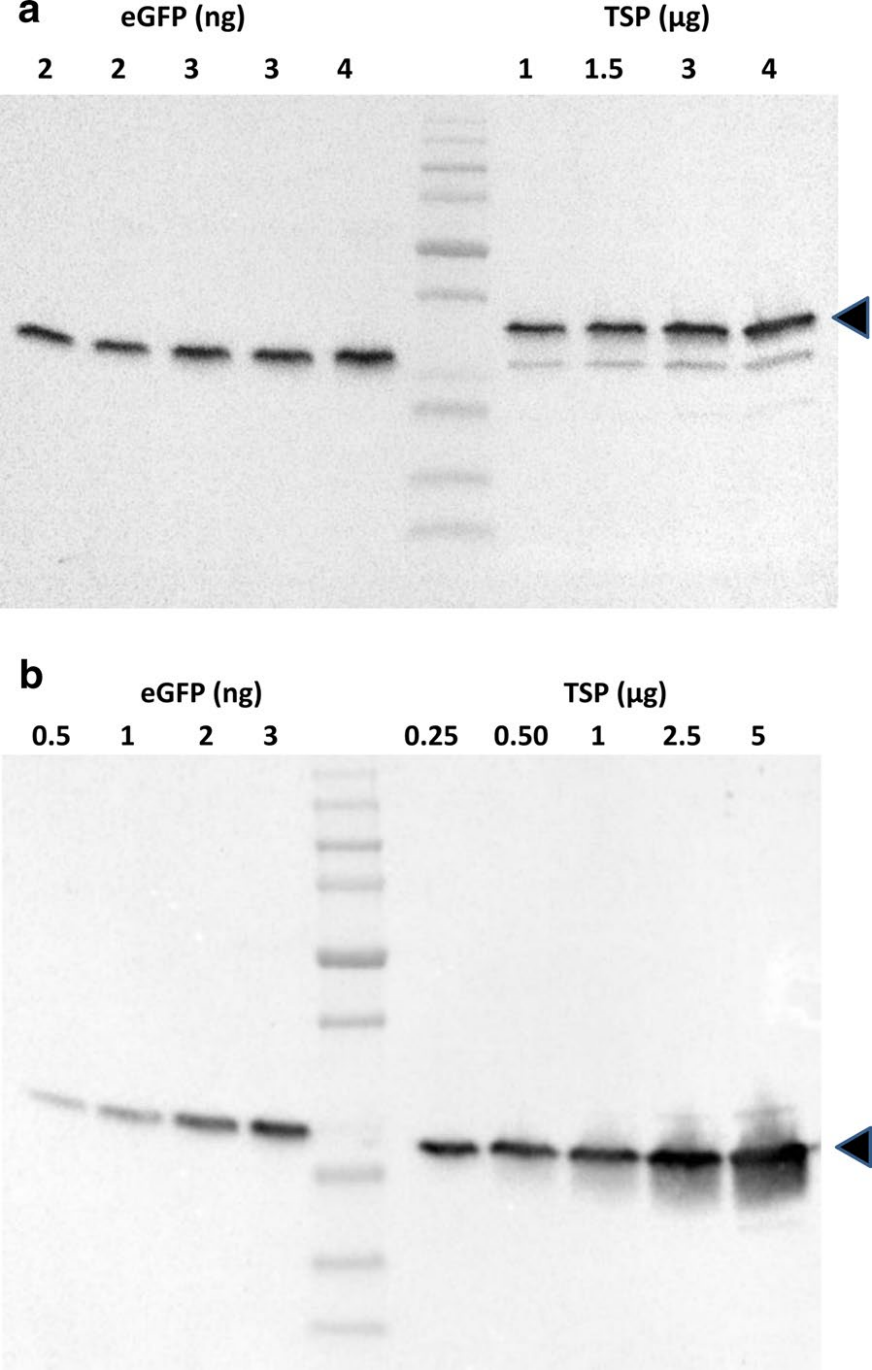
Quantification of eGFP expressed in transgenic *T. pseudonana* (**a**) and *C. cryptica* (**b**) by western blot. PVDF membrane containing transferred GFP standard and total soluble proteins were probed with anti-GFP monoclonal antibodies. BioRad’s ChemiDoc imaging system and Image Lab software were used to detect and quantify chemiluminescent signals from the blot. *Numbers* indicate ng of eGFP and µg of TSP loaded each well. *Arrow heads* indicate recombinant eGFP. The lower band is a native *T. pseudonana* protein with peroxidase activity. eGFP in **a** shift reflects the fused N-terminal SIT sequence

Although eGFP fluorescence is a simple way to estimate expression levels, it does not take into account possible expressed protein that may not be properly folded to enable fluorescence. We therefore quantified respective SIT1 promoter-driven eGFP production in *T. pseudonana* and *C. cryptica* after 24-h silicon-limitation by immunoblotting using different amounts of total soluble protein (TSP) extracted in PBS by sonication. The blot was probed with anti-GFP monoclonal antibodies, and the intensity compared against known amounts of purified recombinant *Aequorea victoria* GFP standard. The eGFP yield was about 1.8 µg per mg total soluble protein (TSP) (0.18% of TSP) in *T. pseudonana* (Fig. 7a) and 12.7 µg per mg TSP (1.3% of TSP) in *C. cryptica* (Fig. 7b).

### Strategies to increase expression level of recombinant proteins: channeling metabolites towards recombinant protein production

**Fig. 8:**
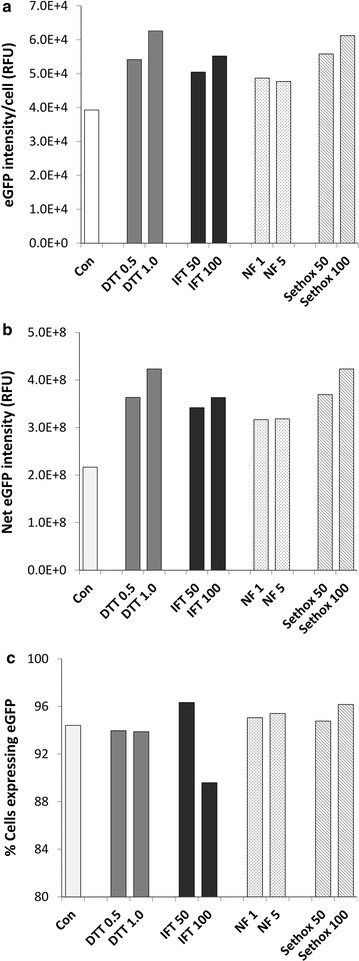
Effect of carotenoid and lipid synthesis inhibitors on recombinant protein expression, under control of SSIP1 promoter, in *T. pseudonana* after 24 h of Si starvation. Expression of eGFP was measured using an imaging flow cytometer (488 nM laser/100 mW with neutral density filter 0.6). *Numbers under bars* indicate concentrations of the inhibitors dithiothreitol (DTT): mM, isoxaflutole (IFT): µg/mL, norflurazon (NF): µM, sethoxydim (Sethox): µM. **a** eGFP intensity/cell; **b** Net eGFP intensity; **c** percentage of cells expressing eGFP. *Error bars* represent standard errors of mean (SEM), n = 20,000. Control sample was without any addition of inhibitors

In addition to selection and optimization of promoters and genes, we hypothesized that inhibition of the functions of chloroplasts, including synthesis of photosynthetic pigments and lipids, will channel metabolic capability and energy towards synthesis of recombinant protein. To test this hypothesis, various inhibitors were added to the media during Si starvation (Table 1). We prescreened the effect of various concentrations of these inhibitors on *T. pseudonana* cell viability, and selected only those concentrations at which cells were able to survive albeit with a slight decrease in cell growth. Figure 8 shows that dithiothreitol (DTT), isoxaflutole (IFT), norflurazon (NF) and sethoxydim increased recombinant eGFP expression level in *T. pseudonana*, though all these treatments also affected normal cell metabolism as evident from the decrease in chlorophyll level (Additional file 3: Figure S3). Depending upon concentration, DTT and sethoxydim were the most efficient eGFP booster with up to twofold increase in net eGFP intensity. IFT and NF were also able to increase eGFP level, but less efficiently than DTT and sethoxydim. We also tested a combination of inhibitors to examine if they synergistically increased eGFP level. Cells were treated with combinations of DTT and sethoxydim. We observed that most of the combinations were too lethal for cells. Thus, the most useful inhibitors to increase recombinant protein were DTT and sethoxydim when used solely.

**Table 1:** List of inhibitors and their mode of action

Inhibitors	Mode of action
Carotenoid biosynthesis inhibitors	
Dithiothreitol (DTT)	Inhibits de-epoxidation violaxanthin to zeaxanthin [41]
Norflurazon	Inhibits phytoene desaturase [41]
Isoxaflutole	Inhibits 4-hydroxyphenylpyruvate dioxygenase (HPPD) [42]
Lipid biosynthesis inhibitors	
Sethoxydim	Inhibits acetyl CoA carboxylase (ACCase) [43]

### Time course of productivity

**Fig. 9:**
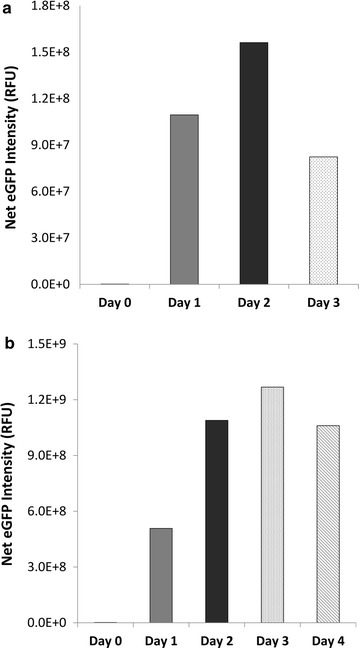
Productivity of recombinant protein expressed in *T. pseudonana* (**a**) and *C. cryptica* (**b**). eGFP was expressed under respective SIT1 control elements and monitored eGFP production under Si starvation by imaging flowcytometry. eGFP was measured with a 488-nm laser with 50 mW power output (**a**) and 100 mW power output after blocking saturation with neutral density filter 1.0 (**b**). *Error bars* represent standard errors of mean (SEM), n = 20,000

We evaluated the relation between initiation of induction by silicon starvation and eGFP yield to determine if there was an optimal time for maximum yield. Cells of *T. pseudonana* and *C. cryptica* expressing eGFP under respective SIT1 control elements were incubated in a 1-L flask under Si deprived conditions and eGFP fluorescence was monitored every 24 h. *T. pseudonana* cells in silicon-deprived medium usually remain healthy only until 48 h, then cell intactness starts to deteriorate. We observed that, for *T. pseudonana*, the net eGFP intensity level increased up to the second day and then declined by day 3 (Fig. 9a). Net eGFP level on day 2 was about 40% higher than day 1. On the other hand, *C. cryptica* cells continued to express eGFP up to 4 days, with maximal productivity on the third day. Net eGFP intensity level on day 2 was twice that on day 1, which further increased up to 150% on day 3 (Fig. 9b).

**Fig. 10:**
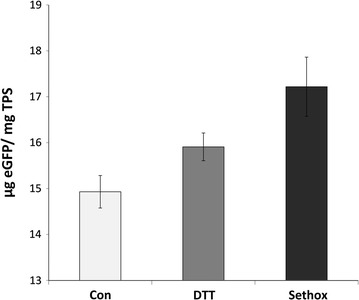
Quantification of eGFP expressed in transgenic *C. cryptica* by ELISA. Total soluble proteins from sonication of cultures harvested on 3rd day of Si starvation were quantified against eGFP standard. *Bars* represent standard deviations (n = 3). Control sample was without any addition of inhibitors

In order to determine the maximum recombinant protein yield with strategies we developed, we further quantified eGFP expression in *C. cryptica* after treatment with DTT or sethoxydim and harvesting on the third day of silicon starvation. ELISA analysis showed that the productivity reached up to 18 µg eGFP per mg TSP (1.8% of TSP), which corresponds to about a 19.8% increase when treated with sethoxydim relative to the control (Fig. 10).

## Discussion

Inducible promoters for versatile and selective induction of recombinant proteins are one of the essential components for high level protein production. Such promoters can minimize detrimental effects on cell growth from overproduction of recombinant proteins or from toxic proteins. Moreover, it is highly desirable to enable expression under favorable metabolic conditions related to the availability of carbon and nitrogen, and during cessation of cell division, where cellular energy and metabolic capability can be channeled into protein synthesis [16, 44]. Only a few inducible algal promoters have been characterized thus far, and these typically rely on induction during growth (reviewed in [14]). Here, we present diatom inducible promoters that overexpress proteins under silicon-limitation conditions. A combination of high level expression coupled to lack of metabolic activities associated with cell division and ancillary processes synergistically increased protein production.

Silicon is a unique requirement for diatoms to make their silicified cell walls, and in nearly all species silicon is required for cell cycle progression [45]. In the absence of silicon, diatom cells cease to divide, but because silicon is not tightly tied into other aspects of cellular metabolism [33], other metabolic activities such as RNA and protein synthesis continue to occur [34]. Evidence for excess energy and carbon availability during silicon limitation is also seen by the ability of diatoms to accumulate large amounts of neutral lipids under these conditions [46–48].

The transport of silicon into diatom cells occurs by two mechanisms, (1) under lower extracellular Si concentrations, uptake is facilitated by silicon transporter (SIT) proteins [49], and (2) under higher extracellular Si concentrations unassisted transmembrane diffusion of the small uncharged silicic acid molecule occurs [50]. SIT transcripts are upregulated in diatoms under silicon limitation [35, 36], presumably as a scavenging response, but because of silicic acid diffusion, the transporters are not needed under silicon-replete conditions, and their transcripts are downregulated. Thus, robust growth occurs without the need for SIT-mediated transport. We have documented a correlation between SIT mRNA and protein levels and cell cycle processes, suggesting that the transporters may play a more dominant role in sensing the absence of silicon, rather than being the major means of import of silicon into the cell under Si-replete conditions [35]. The combination of potentially favorable metabolic and energetic conditions brought about by cell cycle cessation under silicon limiting conditions, and the upregulation of SITs during that time, led us to explore and develop the use of promoters from SITs and similarly regulated genes to drive recombinant protein expression in diatoms.

In *T. pseudonana* we examined a whole genome microarray dataset [39] for genes with expression characteristics similar to the SITs (Fig. 1), and identified several silicon starvation induced proteins. Comparison of RNAseq-based transcript profiles (Fig. 1) and fusion constructs to eGFP (Figs. 2, 3) enabled rapid semi-quantitative screening of the performance of different SSIPs. Transcript levels for TpSIT1 were the second highest in the entire transcriptome under Si limited conditions and were among the least abundant among the SSIP candidates during growth. From that standpoint, the TpSIT1 promoter had the most promising characteristics to drive recombinant protein expression under Si limitation, with minimal expression occurring during growth under Si replete conditions. We also identified a high performance SIT promoter from *C. cryptica* [51], which showed a higher level of eGFP accumulation than those from *T. pseudonana* (Fig. 3). Interestingly, three non-SSIP promoters tested also expressed GFP at higher levels during silicon starvation (Fig. 4). Although other factors may contribute, these results are consistent with an improved ability to synthesize protein due to greater availability of energy and metabolic capacity during silicon starvation division arrest than when cell division is occurring. In a different diatom species, over a 12 h period, total cellular protein levels increased 1.5-fold under Si deplete conditions, compared with 2.5-fold under Si-replete conditions [34]. Considering that protein related to cell division is not needed under Si deplete conditions, the data indicate little detrimental effect on the ability of Si limited cells to synthesize protein.

Our laboratory has pioneered the application of imaging flow cytometry to microalgae, and this approach provides unique insights into population-scale differences in a variety of cellular phenomena [52]. Among the benefits of this approach is the outcome of rigorous statistics by evaluating 10,000 cells in a population, standard error in these analyses is miniscule. The approach enables rapid assessment of detailed characteristics of thousands of cells in a population, and in our analysis, revealed one factor not commonly noted in protein expression. In addition to the mean eGFP fluorescence per cell, which is a proxy for expression level, we showed a substantial variation in the percentage of cells in the population that were expressing at all (Fig. 3). Since this occurs in a clonal population, the most likely explanation is epigenetic effects, perhaps related to differences in intracellular metabolic processes or silicon requirements. It is beyond the scope of the current investigation to explore this aspect of control over expression, however, the data suggest that significant gains in recombinant protein yield could result from overcoming epigenetic effects. Transgene localization is one of the main epigenetic factors [53], but current routine methods of gene transformation, including particle bombardment or electroporation, results in random integration of transgenes. Insertion of transgene at desired safe harbor sites in the genome [54, 55] or use of stable replicating episomes [56] that enable consistent high level expression without negative consequences to other essential genes’ expression is highly desirable. The cloning at pre-determined chromosomal location will be only possible when an efficient homologous recombination method is developed for diatoms. No routine homologous recombination methods have been developed for eukaryotic algae yet except in the haploid eustigmatophyte *Nannochloropsis* sp. [57], but a recently developed CRISPR method for the model microalga *C. reinhardtii*, *T. pseudonana* and *P. tricornutum* is promising [58–60]. Until the availability of routine homologous recombination methods, flow cytometry can be used to select the highest eGFP expression lines, which could minimize both chromosomal location limitations, and epigenetic effects.

One of the strategies to improve recombinant protein yields is to reduce metabolic drain towards unwanted but major competing carbon sinks. Expression levels were consistently higher in the presence of various inhibitors (Fig. 8), consistent with this hypothesis. DTT and sethoxydim resulted in higher eGFP production after Si starvation for 24 h both in *T. pseudonana* and *C. cryptica* (Fig. 10). The resulting increase in recombinant protein could be due to inhibition of violaxanthin de-epoxidase [41], and thus decreased carbon flow towards violaxanthin and other carotenoids, including fucoxanthin, diadinoxanthin, and diatoxanthin [61]. We documented a reduction in chlorophyll fluorescence in the presence of the inhibitors, but it should be considered that was an indirect measure using 488-nm excitation, which is more optimal for excitation of accessory pigments which channel photons into chlorophyll. Similarly, sethoxydim inhibits AcetylCoA carboxylase [43] and should reduce neutral lipid formation, resulting in more carbon and energy available for production of recombinant proteins. Use of chemical inhibitors in a production system could be prohibitively expensive; however, similar phenotypic changes could be engineered into the cell by manipulation of genes involved in the carotenoid or lipid biosynthetic pathways. The knockdown approach using RNAi or antisense RNA [35] expression could be the best solution by inhibiting certain enzyme activities at certain stage of cell growth, for example by controlling expression with the SIT1 promoter. In addition, reducing fucoxanthin, the main accessory pigment in diatoms, could improve light utilization in a high-density culture and lead to an increased overall productivity [62, 63].

Other factors involved in enabling high level expression were examined. First, we found that incorporation of 32 N-terminal amino acids of TpSIT1 (upstream sequence of the first transmembrane domain) increased eGFP level by twofold in comparison to eGFP alone (Fig. 6). The positive effect seen here could be due to more efficient translation initiation related to codon usage (Additional file 2: Figure S2) [17, 64]. On the other hand, it has been shown in bacteria that mRNA structure but not the codon usage at the beginning of genes is the primary factor for efficient translation [65]. If this is true also in diatoms, fusion of native 5′ coding sequence to a codon-optimized heterologous gene should be the method of choice to enhance protein yield. Secondly, we expressed eGFP in a larger diatom, *C. cryptica,* reasoning that larger cells should have a higher proportion of cytoplasmic volume to accumulate recombinant proteins and hence will result into a higher yield in terms per TSP. Expression levels were approximately eightfold higher in *C. cryptica* compared with *T. pseudonana* (Fig. 9), consistent with this. Moreover, productivity of *C. cryptica* continued to increase until 72 h, compared with maximal productivity at 48 h with *T. pseudonana*.

Quantitative evaluation of expression levels using ELISA indicated that we achieved 1.8% of total soluble protein expressing eGFP in *C. cryptica* (Fig. 10). It is important to appreciate that expressed protein yields are highly dependent on the individual protein, and that comparisons of yields between different proteins can be misleading. Moreover, caution should be taken when comparing “typical” protein yields in expression systems, the yield is very much dependent on the particular protein. Comparison of yield of the same protein between systems is valid. In *C. reinhardtii*, chloroplast-expressed yields of GFP were 0.5% TSP [10]. Improvement of nuclear-based expression of GFP in *C. reinhardtii* has been the focus of other work which included a mutagenesis and genetic screen to improve yields [66]. In spite of the sophistication of that approach, yields were only on the order of 0.2% TSP. Our results indicate a 3.6-fold higher yield than that obtained with chloroplast expression and ninefold higher than nuclear expression in *C. reinhardtii.* Given their early stage of development, the ultimate capability of diatom-based expression systems is unknown, however expression of recombinant IgG under growth conditions in the non-silicified diatom *P. tricornutum* yielded 8.7% TSP [67].

A number of improvements could further increase expression efficiency. These include molecular optimization of the transgene such as codon optimization (eGFP described in this work was codon optimized) or interruption of coding sequences by heterologous introns [68, 69]. Protein subcellular localization is also an important factor. Unlike chloroplast-based expression, a potential advantage of nuclear-based expression is that recombinant proteins can be targeted to multiple intracellular compartments. We demonstrated that combined cytoplasmic and chloroplast targeting of a nuclear transgene increased protein yield by 17% in *T. pseudonana* over cytoplasmic targeting alone [40]. Endoplasmic reticulum targeting has proven extremely effective in increasing recombinant protein yields in plants due to improved protein folding and stability [11, 12].

In addition to the fundamental technology for nuclear-based recombinant protein expression in diatoms being established in this and other [22, 23, 67] studies, diatoms provide an attractive system for low-cost, large scale recombinant protein expression. Diatoms are among the most productive unicellular microalgae on the planet, and tend to outcompete other classes of algae for growth [70], indicating an innate efficiency for converting sunlight and nutrients into biomass. Large scale cultivation of diatoms and other algae in the context of biofuels and bioproducts will establish productive and large scale systems from which low cost recombinant protein could be one desirable product [71, 72].

## Methods

### Culture conditions

*Thalassiosira pseudonana* (CCMP1335) and *C. cryptica* (CCMP332) stock cultures were grown in ASW medium [34]. Cultures were grown in 125-mL Erlenmeyer flasks on an orbital shaker under continuous illumination of cool-white fluorescent lamps at 150 μE m^−2^ s^−1^ at 18 °C. For larger volume, cultures were grown in 2 or 8 L glass flasks on magnetic stirrers with air bubbling under continuous light as above.

### Vector construction

#### Gateway expression vectors

Gateway frame B cassette was cloned into EcoRV site of pBluescript generating the destination vector pMHL_71. *T. pseudonana* Gateway™ expression vectors containing eGFP flanked by 5′ and 3′ flanking regions comprised of promoter and terminator of SIT of *T. pseudonana* and *C. cryptica* were constructed by LR clonase recombination of corresponding entry vectors and the destination vector pMHL_71 (Additional file 4: Figure S4). Genes of interest were PCR amplified using primers containing corresponding Gateway att sequences (Additional file 5: Table S1), cloned into the destination vectors using MultiSite Gateway^®^ Pro kit (Life Technologies). Each transformation vector was cotransformed with pMHL_9 expressing *nat1* gene under control of ACCase promoter (received from N. Kroger), which confers resistance to antibiotics nourseothricin.

### Diatom transformation

Expression vectors were introduced into *T. pseudonana* using microparticle bombardment using the Bio-Rad Biolistic PDS-1000/He particle delivery system [13, 19]. Briefly, exponentially grown cells were harvested and 1 × 10^8^ cells were plated in a 5 cm diameter circle in the middle of ASW-agar plate lacking antibiotics. Transformation was then performed by bombarding plasmid DNA coated tungsten beads (M-17, 1.1 µm diameter, BioRad) onto cells plated on the agar plate (1.5% Bacto agar) under vacuum at a distance of 8 cm at 1350 psi. Plates were bombarded twice to obtain higher transformation efficiency. Immediately after bombardment, cells were overlaid with 10 mL of ASW, and placed under light for 24 h. The cells were then plated on NEPC agar containing 100 µg/mL nourseothricin (clonNAT, Werner BioAgents, Germany). Resistant colonies were then picked and transferred into each well of 24 well plates containing 2 mL of ASW with clonNat. Positive clones were then confirmed by PCR using clonNat specific primers as described below.

### Genomic DNA extraction and PCR

Algal cultures grown in 24 well plates were centrifuged (10,000*g*, 2 min) after 1 week, washed once with PBS and resuspended in 25 µL of PBS. The cells were then subjected to three cycles of freeze/defreeze by placing in dry ice for 5 min and heating 2 min at 70 °C. The lysate was then heated for 5 min at 95 °C to denature DNAse. After centrifugation, the supernatants containing genomic DNA were stored at −20 °C until further use. Several antibiotic-resistant clones were screened by growing in 24 wells plates containing antibiotics, followed by confirmation by PCR.

### Western blot

Cells were sonicated twice in ice for 30 s each using a microtip probe (50% duty cycle, Vibra Cell, Sonics & Materials, Inc. CT, USA). The lysate was centrifuged, and the concentration of the supernatant containing total protein extract was measured using Bio-Rad protein assay kit before loading equal amount of protein into each well of BioRad precast gel. PVDF membranes blotted with proteins were incubated with anti-GFP monoclonal antibody (*Living Colors* ^®^ *A.v.* JL-8, Clontech), followed by HRP-conjugated anti-mouse secondary antibodies (Pierce). The blots were then processed using Super signal West Pico kit (Pierce) and exposed on Bio-Rad ChemiDoc system to detect and quantify chemiluminescent signals using Bio-Rad Image Lab 4.1 software.

### Imagestream flowcytometry

Cells transformed with eGFP-tag were analyzed with an ImageStream Imaging Flowcytometer X-100 (Amnis Corp.) using a 100-mW 488-nm laser excitation. At least 20,000 fluorescent as well as brightfield and side scatter cell images per sample were collected at 40× magnifications. Data were then analyzed using image analysis software (IDEAS v5, Amnis Corp).

### Fluorescence microscopy

eGFP expressing cells were photographed with 63×/1.4 objective oil immersion plan APO using Axiovision 4.7.2 software in a Zeiss AxioObserver inverted microscope equipped with an Apotome (Carl Zeiss, NY). The GFP filter set used was Zeiss #38HE (Ex 470/40 nm, FT 495, Em 525/50 nm), and chlorophyll was imaged using filter set #05 (Ex 395–440 nm, FT 460 nm, Em 470 nm LP).

### ELISA

Transgenic diatom cells were sonicated in ice. The total protein extracts were centrifuged, and collected supernatant containing soluble recombinant eGFP. GFP ELISA Kit (Cell Biolabs) was used to quantify eGFP according to manufacturer’s instruction.

## Additional files

**Additional file 1: Figure S1.** Transcript level change after silicon starvation as depicted by microarray analysis. A, FCP; B, rpL41 and NR.

**Additional file 2: Figure S2.** Codon bias of eGFP (upper panel) and 5′ end (96 bp) of TpSIT1 (lower panel) for *T. pseudonana*. The analysis was performed with Rare Codon Calculator (http://www.codons.org/).

**Additional file 3: Figure S3.** Effect of inhibitors dithiothreitol (DTT), isoxaflutole (IFT), norflurazon (NF) and sethoxydim (sethoxy) on cell no. (A) and chlorophyll level (B) measured using Imagestream imaging cytometer.

**Additional file 4: Figure S4.** Schematic diagrams of the Gateway-system based *T. pseudonana* transformation vectors expressing eGFP under the transcriptional control of silicon limitation inducible promoters (SSIPs). SSIP1, TpSIT1 Thaps3_268895; SSIP2, TpSIT2 Thaps3_41392; SSIP3, Thaps3_9619; and SSIP4, CcSIT1 g10780.t1. These vectors were cotransformed with a plasmid vector expressing *Nat1* conferring resistance to the antibiotic nourseothricin.

**Additional file 5: Table S1.** List of primers used.
