# Supplementary figures and images for: Development of a silicon limitation inducible expression system for recombinant protein production in the centric diatoms Thalassiosira pseudonana and Cyclotella cryptica
Source: Microb Cell Fact. 2017 Aug 17;16:145. doi: 10.1186/s12934-017-0760-3 (PMC5561644; doi:10.1186/s12934-017-0760-3)

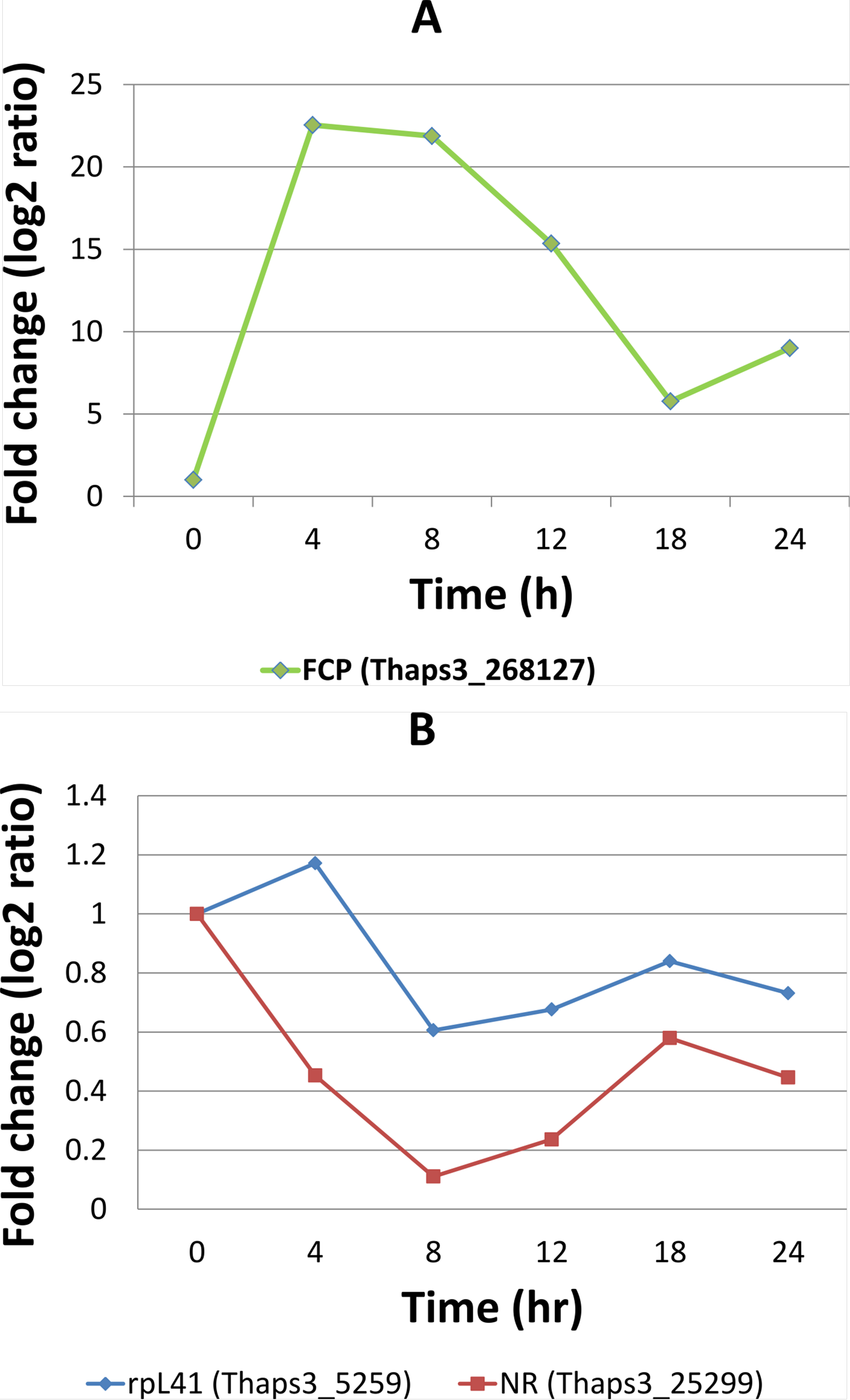

Supplement: Supplementary file 1 — Additional file 1: Figure S1. Transcript level change after silicon starvation as depicted by microarray analysis. A, FCP; B, rpL41 and NR. [file 12934_2017_760_MOESM1_ESM.tif]

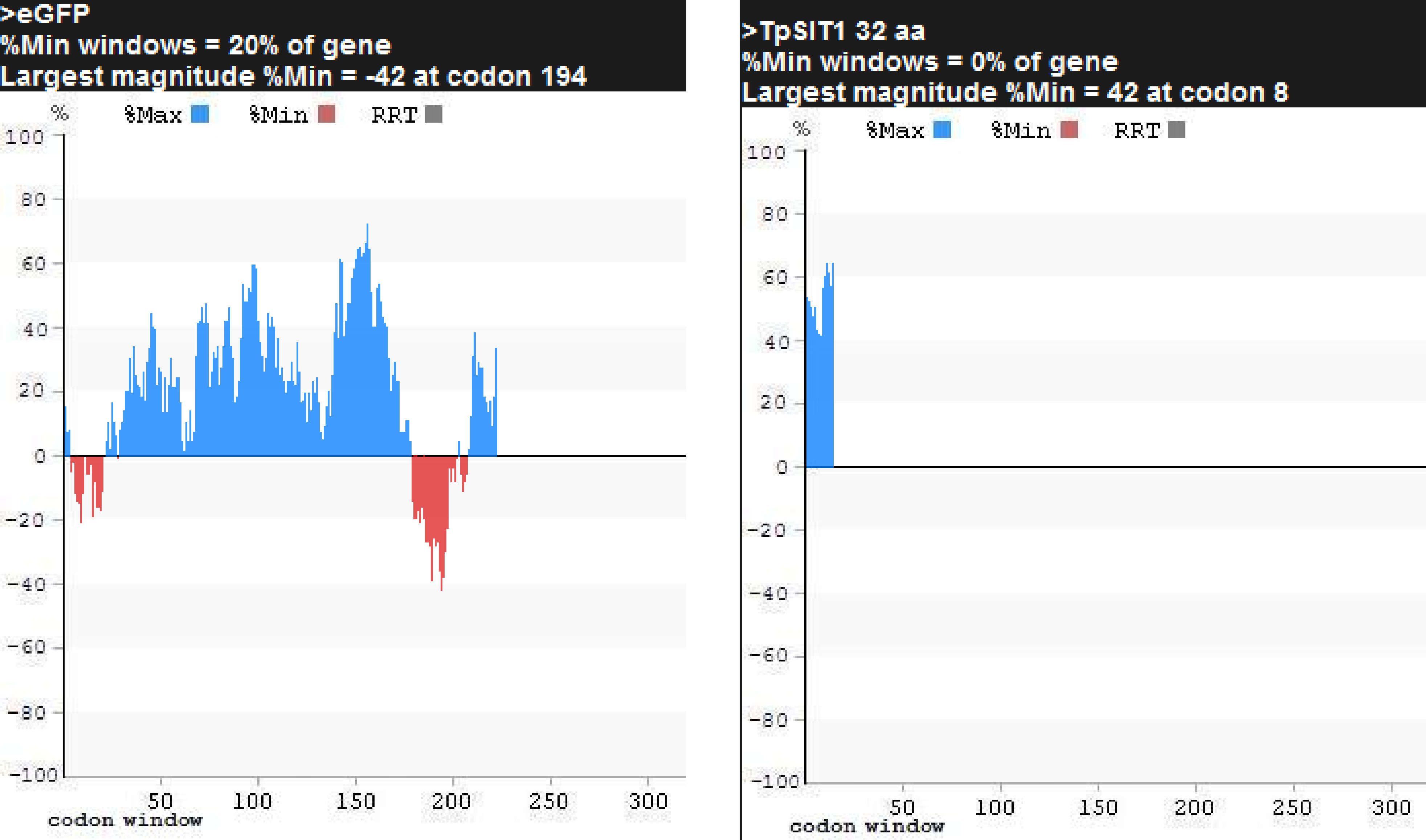

Supplement: Supplementary file 2 — Additional file 2: Figure S2. Codon bias of eGFP (upper panel) and 5′ end (96 bp) of TpSIT1 (lower panel) for T. pseudonana. The analysis was performed with Rare Codon Calculator (http://www.codons.org/). [file 12934_2017_760_MOESM2_ESM.tif]

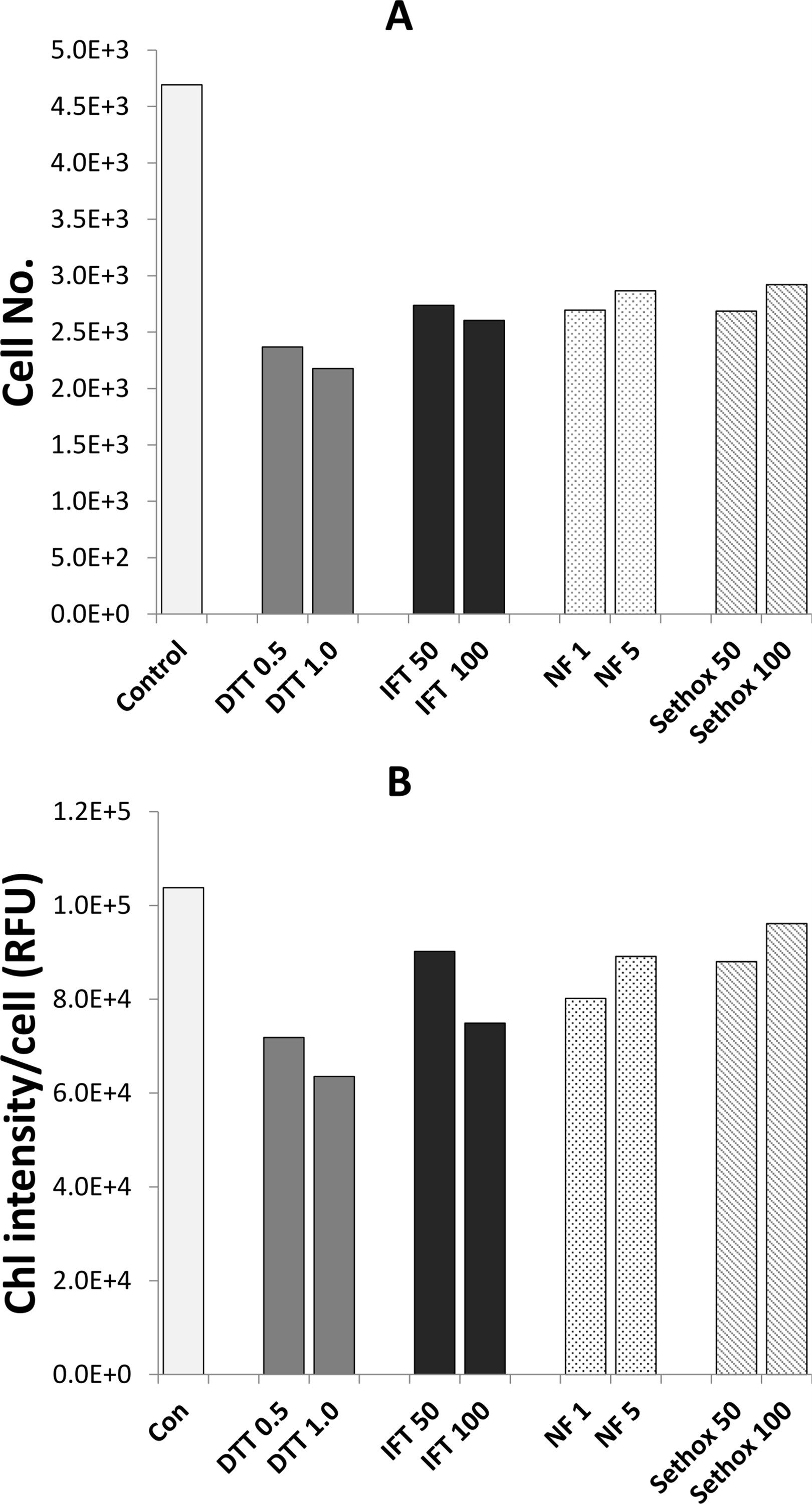

Supplement: Supplementary file 3 — Additional file 3: Figure S3. Effect of inhibitors dithiothreitol (DTT), isoxaflutole (IFT), norflurazon (NF) and sethoxydim (sethoxy) on cell no. (A) and chlorophyll level (B) measured using Imagestream imaging cytometer. [file 12934_2017_760_MOESM3_ESM.tif]

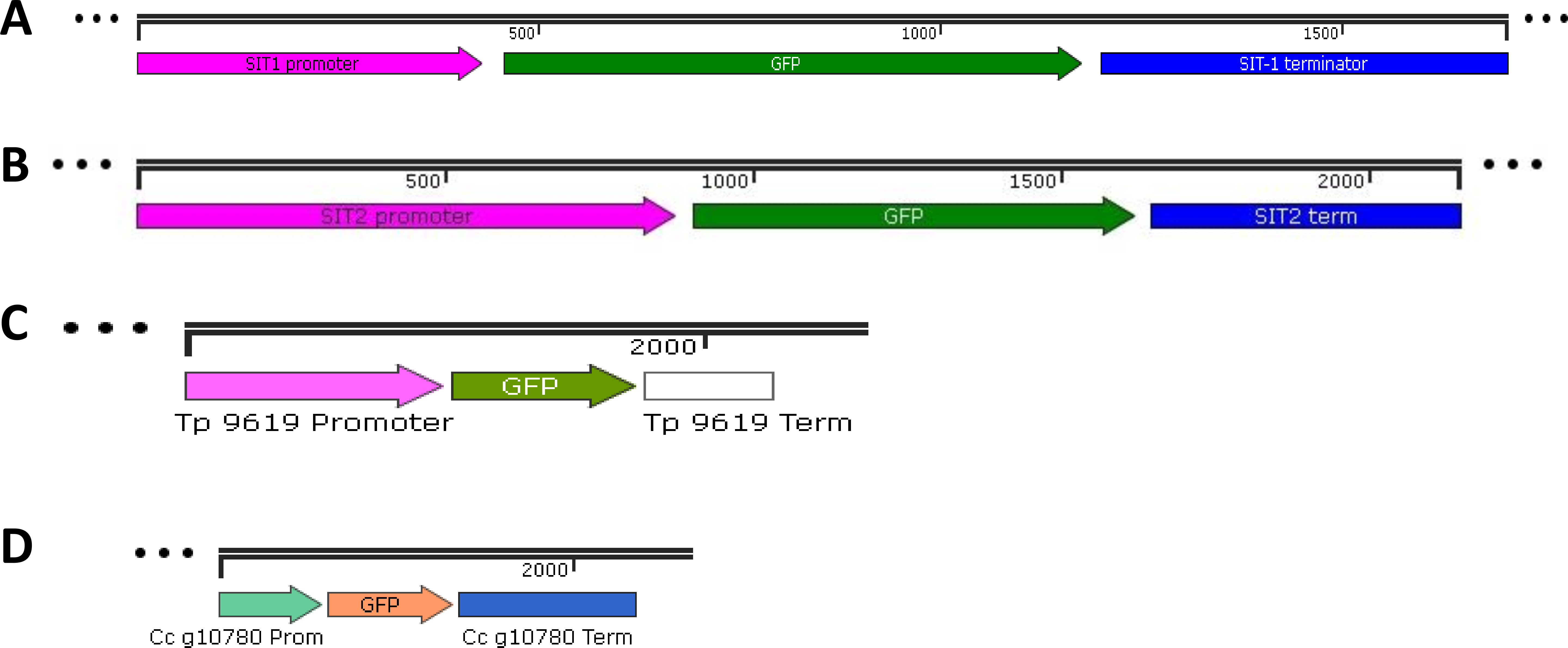

Supplement: Supplementary file 4 — Additional file 4: Figure S4. Schematic diagrams of the Gateway-system based T. pseudonana transformation vectors expressing eGFP under the transcriptional control of silicon limitation inducible promoters (SSIPs). SSIP1, TpSIT1 Thaps3_268895; SSIP2, TpSIT2 Thaps3_41392; SSIP3, Thaps3_9619; and SSIP4, CcSIT1 g10780.t1. These vectors were cotransformed with a plasmid vector expressing Nat1 conferring resistance to the antibiotic nourseothricin. [file 12934_2017_760_MOESM4_ESM.tif]

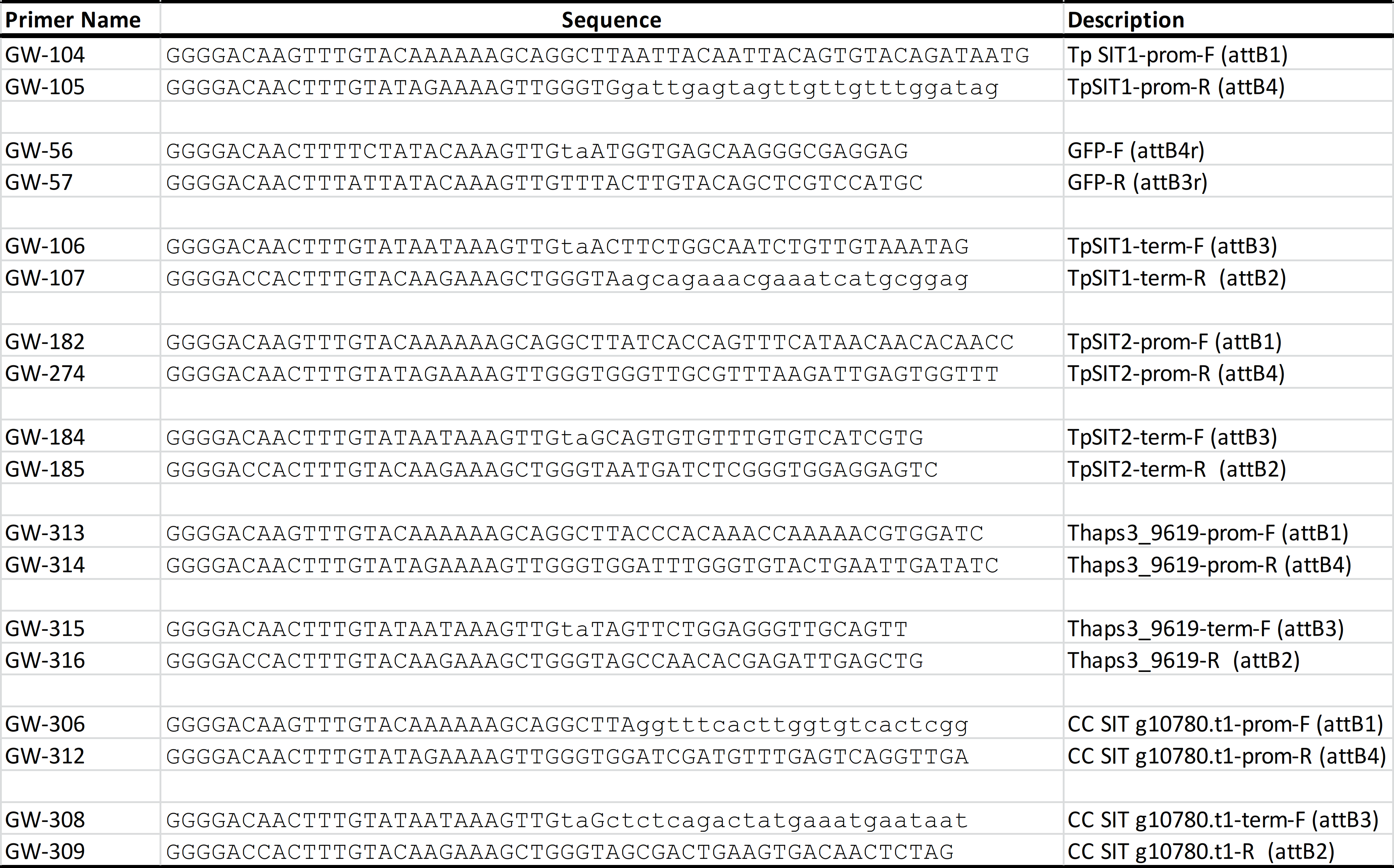

Supplement: Supplementary file 5 — Additional file 5: Table S1. List of primers used. [file 12934_2017_760_MOESM5_ESM.tif]
